# Transfection of *Eimeria mitis* with Yellow Fluorescent Protein as Reporter and the Endogenous Development of the Transgenic Parasite

**DOI:** 10.1371/journal.pone.0114188

**Published:** 2014-12-09

**Authors:** Mei Qin, Xian Yong Liu, Xin Ming Tang, Jing Xia Suo, Ge Ru Tao, Xun Suo

**Affiliations:** 1 State Key Laboratory of Agrobiotechnology, China Agricultural University, Beijing, 100193, China; 2 National Animal Protozoa Laboratory & College of Veterinary Medicine, China Agricultural University, Beijing, 100193, China; 3 Key Laboratory of Animal Epidemiology and Zoonosis of Ministry of Agriculture, China Agricultural University, Beijing, 100193, China; Instituto de Diagnostico y Referencia Epidemiologicos, Mexico

## Abstract

**Background:**

Advancements have been made in the genetic manipulation of apicomplexan parasites. Both the *in vitro* transient and *in vivo* stable transfection of *Eimeria tenella* have been developed successfully. Herein, we report the transient and stable transfection of *Eimeria mitis*.

**Methods and Findings:**

Sporozoites of *E. mitis* transfected with enhanced yellow fluorescent protein (EYFP) expression plasmid were inoculated into chickens via the cloacal route. The recovered fluorescent oocysts were sorted by fluorescence activated cell sorting (FACS) and then passaged 6 generations successively in chickens. The resulting population was analyzed by genome walking and Western blot. The endogenous development of the transgenic *E. mitis* was observed and its reproduction potential was tested. The stable transfection of *E. mitis* was developed. Genome walking confirmed the random integration of plasmid DNA into the genome; while Western blot analysis demonstrated the expression of foreign proteins. Constitutive expression of EYFP was observed in all stages of merogony, gametogony and sporogony. The peak of the transgenic oocyst output was delayed by 24 h and the total oocyst reproduction was reduced by 7-fold when compared to the parental strain.

**Conclusion:**

Stable transfection of *E. mitis* was successfully developed. The expression of foreign antigens in the transgenic parasites will facilitate the development of transgenic *E. mitis* as a vaccine vector.

## Introduction

*Eimeria* spp. are obligate intracellular parasites that can cause avian coccidiosis, which inflicts great economic losses to the poultry industry worldwide [1]–[4]. Currently, chemotherapy is still the main strategy for coccidiosis control. However, development of anticoccidial drug resistance has threatened the economic stability of the poultry industry. Vaccination is an alternative option for coccidiosis control [2], [3], [5], which is effectively used to protect layers and breeders but applied much rarely within the majority broiler sector [4]. Challenges faced in the identification of effective vaccination against pathogens may be matched in the development of optimal, cost-effective, delivery strategies. The tight economic margin and intensive nature of modern poultry production has prompted the development of novel vaccine delivery strategies [6].

Genetic manipulation is a powerful tool for investigating the biology of viruses, bacteria and parasites and for developing novel strategies for the control of infections caused by these pathogens [7], [8]. Transient and stable transfection systems are well established in apicomplexan parasites such as *Toxoplasma gondii* and *Plasmodium falciparum* [9]–[11], and more recently, they have been developed successfully in *Eimeria tenella* [12]–[14]. The establishment of transfection protocols that support stable genetic complementation of *Eimeria* species now encourages the use of these parasites as novel vaccine delivery vehicles. It was demonstrated elsewhere using enhanced yellow fluorescent protein as a model antigen [15] and *Campylobacter jejuni* antigen A(CjaA) as pathogen antigen [16]. *Eimeria mitis*, one of the seven species of chicken coccidia, was first described by Tyzzer in 1929 [17] and considered to be relatively less pathogenic [18]–[21]. Successful genetic complementation of other *Eimeria* species including *Eimeria acervulina*, *Eimeria maxima*, and *Eimeria praecox*, as well as the rat-specific *Eimeria nieschulzi* [22]–[24], support the potential use of less pathogenic species as vectors to develop novel vaccines [4]. Additionally, different intestinal localizations of the seven *Eimeria* species that infect chickens may have great implications in the performance of various *Eimeria* species as vaccine vector. Therefore, we postulated that *E. mitis* parasites could be utilized as an alternative vaccine vehicle for expressing foreign antigens. However, to develop *E. mitis* as a vaccine vector, stable transfection is a prerequisite. We report here transient and stable transfection of *E. mitis* expressing EYFP and avian influenza virus (H9N2) HA1 region fused with chicken IgY Fc fragment (HA1chFc).

## Methods

### Ethics statement

Our research with animals was approved by the Beijing Administration Committee of Laboratory Animals and performed in accordance with the China Agricultural University Institutional Animal Care and Use Committee guidelines.

### Parasites and cell culture

*E. mitis* (Zhuozhou strain) used in the study was maintained by passaging in coccidia-free, 2-5-week-old AA broilers. Procedures for collection, purification and sporulation were carried out as previously described [15], [25], [26].

Madin-Darby bovine kidney (MDBK) cells were cultured in DMEM medium supplemented with fetal bovine serum (10%, v/v) and 1,000 U penicillin/streptomycin in a humidified atmosphere of 5% CO_2_ at 41°C.

### Plasmid construction and transfection of *E. mitis*

**Figure 1:**
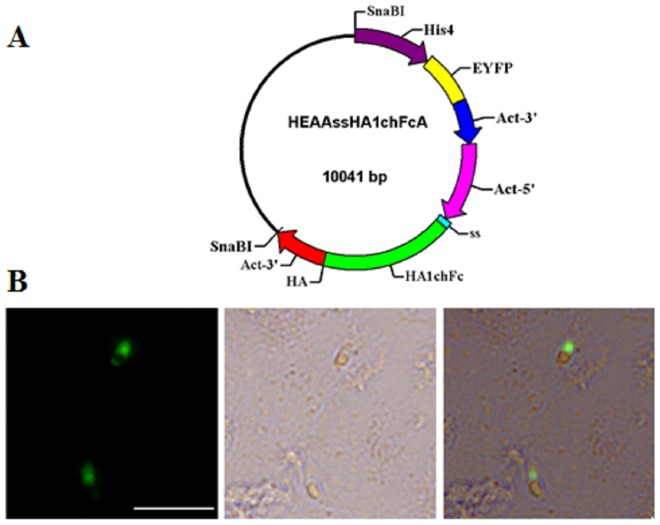
Schematic representation of the plasmid used for the in vitro and in vivo transfection of *E. mitis*. (A) Expression cassettes are shown as colored boxes. The EYFP coding region is flanked by the Histone 4 promoter (His4) and 3′region of actin from *E. tenella*; while the foreign protein region is flanked by the promoter of actin (Act) from *E. tenella*, 3′region of actin and signal sequence (ss, 84 bp) derived from dense granule protein 8 (GRA8) of *T. gondii*. (B) Sporozoites transfected with pHEAAssHA1chFcA in MDBK cell. Bar = 20 µm.

The double expression-cassette plasmid, pH4-EYFP/ACT-HA1chFc-ACT (pHEAAssHA1chFcA), was constructed based on pH4-EYFP/ACT-IMP1-RFP-ACT[26], with the IMP1-RFP fragment replaced by avian influenza virus (H9N2) HA1 region fused with chicken IgY Fc fragment (HA1chFc) (Fig. 1A). The plasmid DNA was linearized by SnaBI restriction enzyme, which released the two expression cassettes from the backbone of plasmid (Fig. 1A).

*E. mitis* sporozoites were freshly purified through diethylaminoethyl-52 cellulose (DE-52 cellulose) column and re-suspended in cytomix buffer supplemented with 2 mM ATP and 5 mM glutathione [27], [28]. Parasite transfection was conducted using REMI strategy and the Amaxa Nucleofector system as described previously [13], [29]. For the transient transfection assay, nucleofected sporozoites were inoculated onto confluent MDBK cells. For the *in vivo* assay, two million of nucleofected sporozoites were inoculated into 7-day-old chickens via the cloacal route [13]. Oocysts were collected from feces 5–8 days post-inoculation. The oocysts expressing EYFP (rE.mi) were sorted from the progeny population by the MoFlo Cell Sorter (Dako-Cytomation, Fort Collins, CO) on the single-cell mode and inoculated into coccidian-free chickens for the propagation of next generation [14]. This process of sorting and propagating was successively carried out for 7 times.

### Genomic DNA analysis of transgenic *E. mitis*

Genomic DNA was isolated from sporulated transgenic oocysts by phenol/chloroform extraction and ethanol precipitation [30]. The DNA pellet was dissolved in distilled water with 0.4 mg/ml RNase H for high-quality genome DNA. To detect the presence of EYFP and chimeric HA1chFc fragments in the transgenic parasites, PCR verification was performed with primer pairs for HA1chFc (5′-CTACTACTGGAGCGTGCTGAAGCC-3′ and 5′-GTACTCCTTGCCTGCCTGCTTCTG-3′) and EYFP (5′-ATGGTGAGCAAGGGCGAGGA-3′ and 5′-AAGCTTCTTGTACAGCTCGT-3′). Genomic DNA of wild type *E. mitis* was used as control template.

To validate the integration of the DNA fragment into the genome of transgenic *E. mitis*, the flanking sequences to the 5′ of the integrated DNA were detected using a Genome Walking Kit (Takara, Dalian, China). Specific reverse primers, SP1 (5′-GCTTGCAGCACTTCAGACACTCAA-3′), SP2 (5′-AAAGACAGAAGTGCCAGCAGCAG3-′) and SP3 (5′-CTGCAACATTCAGTGACTTAGCCG3-′) were designed according to His4 promoter sequence following kit instructions. The forward primers AP1, AP2, AP3 and AP4 were supplied in the Genome Walking Kit. PCR amplification was carried out as follows: first PCR at 94°C for 1 min, 98°C for 1 min, 94°Cfor 30 s, 65°C for 1 min, and 72°C for 2 min for 5 cycles; 94°Cfor 30 s, 25°C for 3 min, and 72°Cfor 2 min; 94°Cfor 30 s, 65°Cfor 1 min, and 72°C for 2 min; 94°Cfor 30 s, 65°C for 1 min, and 72°C for 2 min for 15 cycles; 94°C for 30 s, 44°C for 1 min,72°C for 2 min, and 72°C for 10 min. The second and third PCR reactions each consisted of 94°C for 30 s, 65°C for 1 min, and 72°C for 2 min; 94°C for 30 s, 65°C for 1 min, and 72°C for 2 min;94°C for 30 s, 44°C for 1 min, and 72°C for 2 min for 15 cycles; and an extension at 72°C for 10 min. The third round PCR products selected were purified and cloned into pEASY-T1-simple vector (TransGen Biotech) and confirmed by DNA sequencing. The resulting putative sequences were then analyzed by DNAStar7.0 software, and the integration sites in the genome were identified by performing a BLAST search in the *E. mitis* DB database [31].

### Western blot analysis

Protein extraction and Western blot analysis for rE.mi was carried out as previoulsly described [30]. Briefly, soluble protein extracted from sporulated oocysts was resolved by SDS-PAGE and transferred onto PVDF membranes with duplicate. For the detection of HA1 in fused HA1chFc protein, one membrane was probed with mouse polyclonal anti-HA1 antibody as the primary antibody and followed by HRP-conjugated goat anti -mouse IgG; while another membrane was probed with HRP-conjugated goat anti-chicken IgY Fc directly for the detection of chFc in the fusion protein.

### Observation of endogenous development of transgenic *E. mitis*

Eighteen 10-day-old AA broilers were orally infected with 1×10^6^ rE.mi oocysts. From 24 h to 130 h post inoculation (p.i.), two birds were killed for necropsy 12 hours at intervals. Fresh smears were made from pieces of various tissues from the intestines and visualized using a confocal laser scanning microscopy (SP5, Leica, Germany) for the detection of YFP-expressing parasites.

### Reproduction test of rE.mi

To assess the reproduction of the transgenic parasites, two groups of 4 chickens were infected with 1000 sporulated oocysts of rE.mi and its parental strain, respectively. Fecal samples were collected every 24 h between 5 d and 11 d after infection. Oocysts shed in the feces was determined using McMaster egg counting chamber [15], [32].

## Results

### Expression of YFP in rE.mi

At 24 h p.i., sporozoites transfected with the plasmid pHEAAssHA1chFcA were observed expressing EYFP. Fluorescence was predominantly located within the nucleus of rE.mi (Fig. 1B), as EYFP was under the control of the nuclear localization sequence from *E. tenella* Histone 4[23].

**Figure 2:**
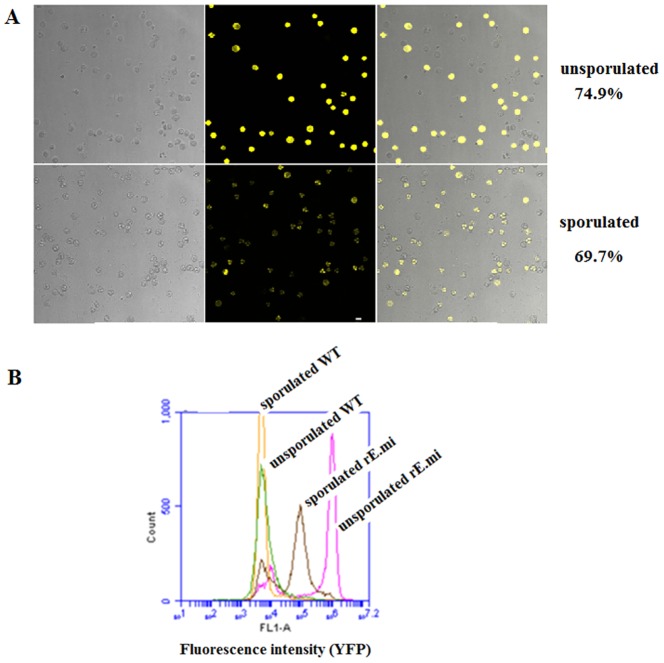
Validation of rE.mi stably expressing fused protein and EYFP molecule. (A) Fluorescent images of the seventh passage of unsporulated and sporulated oocysts of rE.mi;(B) Fluorescence intensity of unsporulated and sporulated oocysts of rE.mi. Flow cytometry was performed with a FACS Calibur analyzer (BD Biosciences, USA). Data was analyzed using Cell Questpro Software (BD Biosciences, USA).

After the first *in vivo* propagation and sorting, the ratio of fluorescent sporulated oocysts was 0.19% in the population. Subsequent serial passages using FACS sorting resulted in gradual increase of EYFP-expressing sporulated oocysts, up to 68.9% at the seventh passage (Table 1). Interestingly, more unsporulated oocysts (74.9%) expressed EYFP as compared with sporulated oocysts (69.7%) (Fig. 2A). This phenomenon was due to the decrease of fluorescence intensity of the oocysts during sporulation (Fig. 2A and 2B).

**Table 1:** Transfection efficiency of rE.mi of different passages.

			Percentage (%)				
	1st	2nd	3rd	4th	5th	6th	7th
rE.mi	0.1	5	32	30	50	69.7	68.9

### Validation of the stable transfection of *E. mitis*

**Figure 3:**
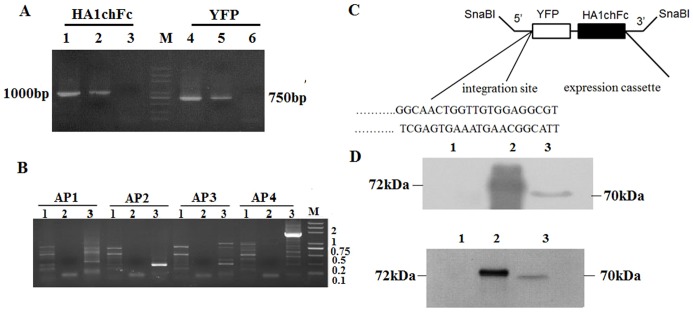
Genome analyses of inserted DNA fragments in the rE.mi population. (A) Detection of HA1chFc and EYFP fragments by PCR. Lane 1 and 4, PCR products from plasmid pHEAAssHA1chFcA (positive control); lanes 2 and 5, DNA extracted from rE.mi; lanes 3 and 5, DNA extracted from wild type of *E. mitis* (negative control); M, DL plus 2000 molecular weight ladder. (B) Gel electrophoresis of amplified products after three nested genome walking PCRs. 1–3 represent rounds of PCR, AP1-4, random primers from genome walking kit. M, DL plus 2000 marker. (C) Integrated sites of plasmid expression cassettes into *E. mitis* genome by Genome walking. (D) Detection of recombined HA1chFc protein by Western blotting with mouse HA1 protein polyclonal antibody (upper) or chicken IgY Fc antibody (below). Lane 1, protein from wild type of *E. mitis* (negative control); lane 2, purified HA1chFc protein expressed in *E.coli* (positive control); lane 3, protein extracted from rE.mi.

The presence of EYFP and HA1chFc fragments in the transgenic parasites was confirmed by PCR detection of genomic DNA (Fig. 3A). The 5′ integration sites were confirmed by sequence analysis of the recovered fragments of genome walking events (Fig. 3B and C). Sequence analysis showed that the constructs had integrated into the genome of *E. mitis*, with two integration sites (one in Emh_scaff11823 and the other in Emh_scaff14114). As the sites with inserted integration have not been annotated yet, we can not clarify the location of plasmid integration.

When antibodies against both HA1 and chFc were applied to the immunoblotting assay, bands of the same size to the recombinant HA1chFc protein were detected, demonstrating the expression of both HA1 and chFc in rE.mi (Fig. 3D).

### Transgenic *E. mitis* constitutively expressing EYFP throughout the life cycle

**Figure 4:**
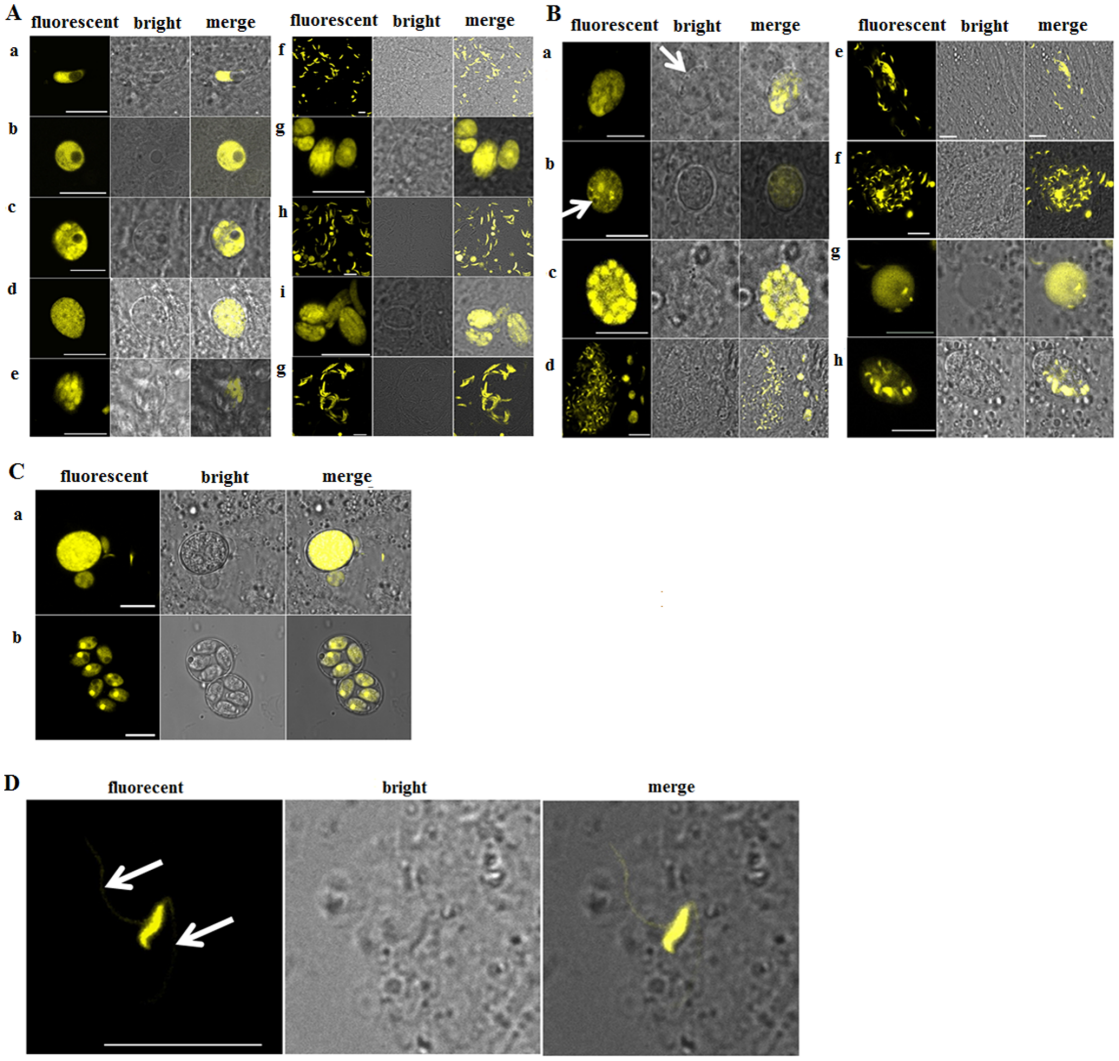
Transfected *E. mitis* constitutively expressed EYFP molecule throughout the life cycle. (A) Merogony stage; a-b, trophozoites; c, trophozoites developing into merozont; d, immature merozont; e-j, merozonts and merozoites; (B) Gamogony stage; a, macrogamete at early stage; b, mature macrogamete; c-f, microgametogenesis; g, fertilization of microgametes and macrogametes; h, zygotes; (C) Sporogony stage. a, unsporulated oocysts; b, sporulated oocysts. (D) Flagella of microgamete. Bar = 10 µm. The data represented one of three independent experiments with similar results.

To investigate whether EYFP was constitutively expressed, we observed both endogenous and exogenous development of transgenic *E. mitis* using a confocal laser scanning microscopy. Sporozoites were fluorescent at 24–29 h p.i. (Fig. 4 A a), but their refractile globules were not (data not shown). Fluorescent trophozoites (Fig. 4A a-c), meronts and merozoites (Fig. 3A d-j) were observed in tissue smear samples between 24 h and 88 h p.i.. At 120 h, we also found fluorescenct macrogametes (Fig. 4B a and b), microgametocytes (Fig. 4B c) and microgametes (Fig. 4B d-f). Interestingly, we found microgamets were attached to macrogamets or possible zygotes (Fig. 4B g and h). Oocysts unsporulated and sporulated were both fluorescent, with the nucleus of sporozoites showing higher intensity of fluorescence (Fig. 4C).

To take the advantage of the EYFP reporter, we observed some detailed morphology of microgametogenesis. We found the nuclei were peripherally located in the mature microgametocytes (Fig. 4 B c), and microgametes subsequently developed (Fig. 4 B d-f), with two flagella arising from the basal body on the mature microgamete (Fig. 4 D, arrows indicated).

### Reproduction of rE.mi

**Figure 5:**
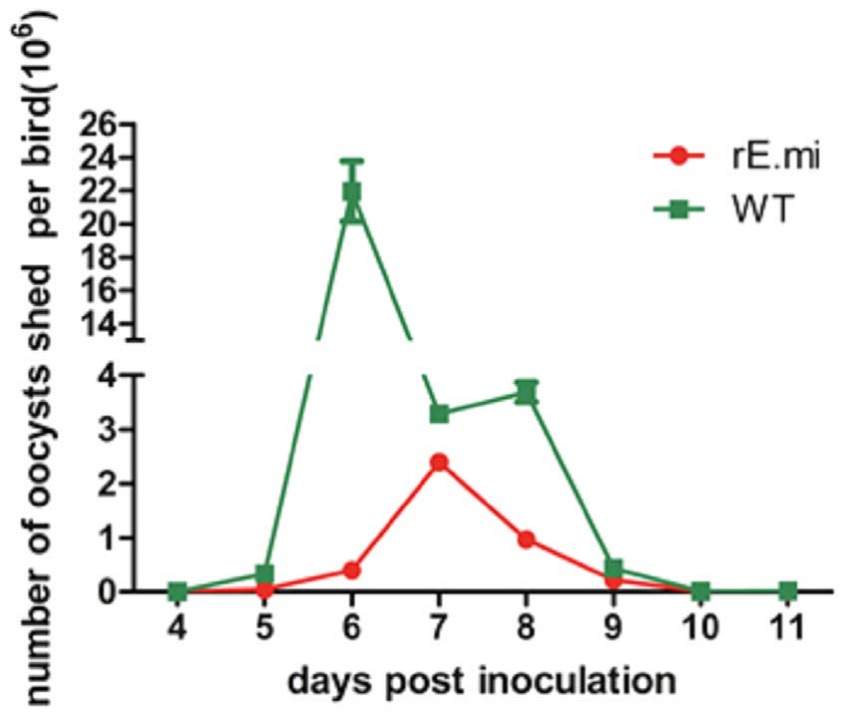
Comparison of the oocyst shedding pattern of rE.mi with that of the parent strain. One group of four chickens were infected with 1000 rE.mi, another group received 1000 parent parasites. Oocyst shedding was measured every 24 h between 5d and 11d post infection. The data represented one of two independent experiments with similar results and were expressed as the mean ± SD.

Oocysts shedding peak of rE.mi was delayed 24 h and the total of oocyst output was reduced 7-fold compared with the parent strain. The fecundity potential of transgenic *E. mitis* was significantly smaller than that of the parent strain (0.67×10^6^/bird vs 4.7×10^6^/bird) (Fig. 5).

## Discussion

Recently, genetic manipulation of *E. tenella* was proved to be feasible and both transient and stable transfection systems in *E. tenella* were established [12], [14]–[16], [30]. Here, we first describe transient and stable transfection of *E. mitis*, another species of chicken coccidia. We found linearized plasmid with regulatory sequences derived from *E. tenella* was integrated and transfected genes were expressed. And interestingly, fluorescent parasites were useful for the observation of details of the life cycle. The expression of EYFP and HA1chFc in transgenic *E. mitis* proved that the regulatory sequences derived from *E. tenella* were functional in *E. mitis*. This finding is in accordance with a previous report, which demonstrated that *E. tenella* histone 4 promoter sequence could function effectively in the transient transfection of *E. maxima*, *E. acervulina* and *E. praecox* [24].

Strategies used for the transfection of *E. tenella*, such as REMI transfection,nucleofection of sporozoites and cloaca inoculation [13], [14], [33], are very helpful for the transfection of *E. mitis*. Using the above strategies, we obtained stably transfected *E. mitis* via FACS of EYFP-expressing oocysts through 7 successive passages. Genetic analysis of rE.mi revealed that two integrations occurred in the genome of the transgenic population, and these integrations may have their implications in some of the observed phenotype changes such as oocyst shedding peak days and reduced fecundity, either through disruption of host genes and/or expression of foreign genes [14]. Establishment of a clonal population of transgenic parasites is of great importance for the investigation of their phenotypes, like influence on fecundity and oocyst output peak owning to difference in integration sites. As *in vitro* cultivation of *E. mitis* is not successful, we tried to establish a clonal population of transgenic *E. mitis* through inoculation of single sporocyst, but failed. Difficulty in establishment of clonal populations of rE.mi expressing HA1chFc may be due to a reduced fecundity [34] or other unknown factors.

The constitutive expression of EYFP in transgenic *E. mitis* made it convenient to observe the morphology of the parasites. The peripheral location of the nuclei of mature microgametocytes in microgametogenesis revealed that microgametes were directly differentiated along with the migrating of nuclei to peripheral location. The differentiation of microgametes of *E. mitis* resembles previous reports on *Eimeria auburnensis*, *Eimeria perforans*, and *E. tenella* through electron microscopy observation [35]. Additionally, we found that two flagella were present in an *E. mitis* microgamete in many smears, resembling *Eimeria brunetti, Eimeria magna* and *T. gondii* [36]–[38], but unlike microgametes of *E. nieschulzi*, *Eimeria falciformis*, *E. perforans*, *E. maxima* and *E. acervulina* which have three flagella [35], [39]–[41].

Transgenic *E. tenella* expressing yellow fluorescent protein as a model antigen can stimulate a range of humoral and cell mediated immune responses in the chicken [15]. Immunized chickens with transgenic *E. tenella* expressing CjaA as pathogen antigen was found to stimulate an anti- CjaA immune response and reduce *Campylobacter jejuni* colonization in the gut by between 86% and 91% compared with controls [16]. However, commercialization of transgenic *Eimeria* parasites as a vaccine vector requests their stimulating higher protective immunity. The quantity of foreign proteins expressed by transgenic *Eimeira* has great effect on the magnitude of protective immunity to the pathogens from which foreign antigens are derived. The relatively low expression level of foreign proteins in transgenic *Eimeira* lead us to choose a less pathogenic *E. mitis* as a promising vaccine vector, because we could use higher immunization dosage of transgenic *E. mitis* as an alternative way to increase the quantity of foreign antigens. Research is being conducted to test our rational.

## Conclusion

In summary, we successfully developed the *in vivo* transfection of *E. mitis* and obtained a transgenic *E. mitis* population. The establishment of transgenic *E. mitis* is a positive step for the development of transgenic *E. mitis*-based vaccine vector for the control of other major avian diseases.
